# Nanostructured Lipid Carriers Containing Norfloxacin and 2-Aminothiophene Derivative Reduces Fluoroquinolone Resistance in Multidrug-Resistant *Staphylococcus aureus* Strains by Efflux Pump Inhibition

**DOI:** 10.3390/pharmaceutics18020183

**Published:** 2026-01-30

**Authors:** Aléxia Gonçalves Dias, Izabele de Souza Araújo, Rodrigo Santos Aquino de Araújo, Malu Maria Lucas dos Reis, Cícera Datiane de Morais Oliveira Tintino, Saulo Relison Tintino, Gildênia Alves de Araújo, Priscilla Augusta de Sousa Fernandes, Henrique Douglas Melo Coutinho, Elquio Eleamen Oliveira, Francisco Jaime Bezerra Mendonça-Junior

**Affiliations:** 1 Postgraduate Program in Natural and Synthetic Bioactive Products, Federal University of Paraíba, João Pessoa 58051-900, Brazil; alexiajgdias@gmail.com (A.G.D.); izabele.araaujo@gmail.com (I.d.S.A.); prisciasf@gmail.com (P.A.d.S.F.); 2 Laboratory of Synthesis and Drug Delivery, Department of Biological Sciences, State University of Paraíba, João Pessoa 58071-160, Brazil; rodrigobiologojp@gmail.com (R.S.A.d.A.); malureisduarte@gmail.com (M.M.L.d.R.); franciscojaime@servidor.uepb.edu.br (F.J.B.M.-J.); 3 Laboratory of Microbiology and Molecular Biology, Universidade Regional do Cariri, Crato 63105-000, Brazil; datianemorais@gmail.com (C.D.d.M.O.T.); saulo.tintino@urca.br (S.R.T.); gildenia.araujo@urca.br (G.A.d.A.); hdmcoutinho@gmail.com (H.D.M.C.)

**Keywords:** 2-amino-thiophene derivative, efflux pump inhibitors, multidrug resistance, nanostructured lipid carriers

## Abstract

**Background/Objectives:** Multidrug resistance (MDR) remains a critical global public health concern, compromising the efficacy of currently available antibiotics. As the development of new antibiotics offers limited long-term solutions, alternative approaches such as efflux pump inhibition have gained attention. This study reports the development of nanostructured lipid carriers (NLCs) co-loaded with Norfloxacin (NOR) and the efflux pump inhibitor 2-amino-thiophen-6CN-Ethyl, to modulate NOR activity against resistant *Staphylococcus aureus* strains overexpressing efflux pump genes. **Methods:** NLCs were produced via the hot emulsion method followed by sonication. The formulations were characterized for encapsulation efficiency (EE%), particle size, polydispersity index (PDI), zeta potential, X-ray diffraction (XRD), infrared spectroscopy (FTIR), differential scanning calorimetry (DSC), scanning electron microscopy (SEM), in vitro release kinetics, and stability. Antibacterial activity was evaluated against *S. aureus* 1199B and K2068 strains. **Results:** The NLC formulation containing norfloxacin and 6CN-Ethyl (NLC10NOR + 106CN) demonstrated high EE% for both compounds (99.50% for 6CN-Ethyl and 90.91% for NOR) and physicochemical stability over 60 days (particle size < 255 nm, PDI < 0.3, zeta potential < −20 mV). Structural analyses confirmed amorphization and effective encapsulation of the active constituents. Antibacterial assays showed that NLC10NOR + 106CN significantly increased NOR activity compared to the free drug and physical mixture; the effect in *1199B* was notably superior to the NOR + CCCP (carbonyl cyanide *m*-chlorophenylhydrazone) combination. **Conclusions:** These findings highlight the potential of NLC-based co-delivery systems as innovative strategies to overcome bacterial resistance, particularly through efflux pump inhibition enhancing antibiotic efficacy.

## 1. Introduction

Bacterial resistance is a growing global problem, regardless of social class and access to healthcare [1]. *Staphylococcus aureus*, a Gram-positive bacterium, is among the most resistant bacteria and frequently associated with serious infections, especially when it reaches the bloodstream. Infections caused by methicillin-resistant strains, called methicillin-resistant *Staphylococcus aureus* (MRSA), have a mortality rate up to three times higher than that observed for sensitive strains [2,3]. This high antimicrobial resistance justifies the inclusion of *S. aureus* in the ESKAPE group, which includes the bacteria *Enterococcus faecium*, *S. aureus*, *Klebsiella pneumoniae*, *Acinetobacter baumannii*, *Pseudomonas aeruginosa*, and species of the *Enterobacter* genus. This group includes pathogens associated with serious nosocomial infections and limited treatment due to resistance to multiple antibiotics [4].

One of the main mechanisms involved in bacterial resistance refers to overexpression of transmembrane proteins called efflux pumps. These proteins are classified as active transport systems, which are responsible for extruding compounds from the cytoplasm to the extracellular environment, including several classes of antimicrobial agents [1].

The existence of six main families of efflux pumps is described in *S. aureus*, with the Major Facilitator Superfamily (MFS) which expresses the NorA pump, and the Multidrug and Toxic Compound Extrusion (MATE), of which the MepA pump is a part, standing out. The activity of these pumps contributes to multidrug resistance, including fluoroquinolone antibiotics such as ciprofloxacin and norfloxacin [5,6].

**Figure 1:**
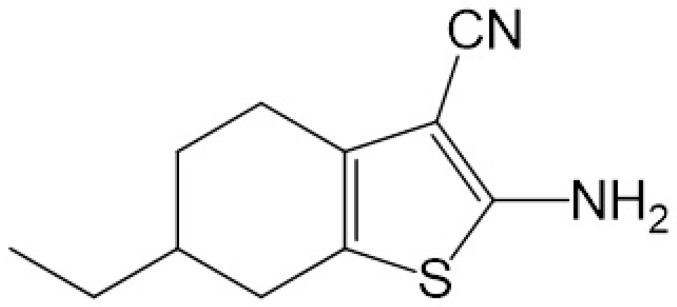
Structure of the 2-aminothiophene derivative 6CN-Ethyl.

Thiophenic derivatives constitute a class of heterocyclic compounds of interest in pharmacological research due to their diverse biological properties, including antiprotozoal, antifungal, and antiproliferative activities, and as antimicrobial resistance modulator agents through inhibition of efflux pumps [7,8,9,10]. However, the low aqueous solubility of these compounds represents a significant obstacle to their bioavailability [11,12]. In a study conducted by our research group, the inhibitory activity of a series of 2-aminothiophenic derivatives on the NorA and MepA efflux pumps in *S. aureus* was evaluated. Among the compounds tested, the derivative 2-amino-6-Ethyl-4,5,6,7-tetrahydrobenzo[b]thiophene-3-carbonitrile (6CN-Ethyl) has been shown to be effective in restoring susceptibility to the fluoroquinolones norfloxacin and ciprofloxacin in resistant strains due to overexpression of these efflux pumps [13] (Figure 1). In this context, efflux pump inhibitors act by preventing the extrusion of substances considered toxic by bacteria, such as antibiotics, contributing to reverse or reduce bacterial resistance, while also contributing to increasing the shelf life of known antibiotics [14].

Considering the aqueous solubility limitations of thiophene derivatives, strategies based on controlled-release systems can be employed. Among these approaches, encapsulation in lipid nanoparticles stands out as a promising alternative, as it enhances apparent solubility and consequently improves the pharmacokinetic profile and biological activity of these compounds [15]. In particular, nanostructured lipid carriers (NLCs) consisting of a hybrid matrix of solid and liquid lipids offer substantial advantages over first-generation lipid systems, such as greater encapsulation capacity, improved stability, and reduced premature drug expulsion during storage [16]. Furthermore, these carriers enable more efficient control of active ingredient release, exhibit biodegradability, ease of handling, economic viability, and excellent physical and chemical stability, representing a promising approach for delivering bioactive compounds with low solubility [17,18].

In this work, NLCs containing the antibiotic norfloxacin and the 6CN-Ethyl efflux pump inhibitor were developed, aiming to enhance the antibacterial efficacy of norfloxacin against *Staphylococcus aureus 1199B* and *K2068* strains, which present resistance to fluoroquinolones due to the overexpression of NorA and MepA efflux pumps, respectively.

## 2. Materials and Methods

### 2.1. Materials

Soy lecithin (Lipoid^®^ S-100) was purchased from Lipoid GmbH (Ludwigshafen am Rhein, Germany), and D-(+)-Trehalose dihydrate 99% was purchased from Alfa Aesar (Ward Hill, MA, USA). Absolute ethyl alcohol P.A. was supplied by Dinâmica Química (Indaiatuba, Brazil). Oleic acid, stearic acid, Compritol^®^ 888 ATO, Precirol^®^ ATO, Gelucire^®^ 44/14, Gelucire^®^ 43/01, Gelucire^®^ 50/13, Miglyol^®^, Capryol^®^, Labrafac^®^ and Transcutol^®^ were purchased from Gattefossé (Saint-Priest, France). Tween^®^ 80 was obtained from P.A. Sol. Tech (Pedranópolis, Brazil). Pluronic^®^F68, Polyvinyl alcohol (PVA) and norfloxacin were purchased by Sigma-Aldrich (St. Louis, MO, USA). Dialysis membrane MWCO 12 000 (Sigma Aldrich, Jurubatuba, Brazil). Compound 6CN-Ethyl was resynthesized in the Laboratory of Synthesis and Drug Delivery, following a methodology previously described by Mendonça Júnior et al. (2011) [19]. Type II pure water was obtained using a 5-stage reverse osmosis system with a flow rate of 12 L·h^−1^, MARTE^®^ ORM-15, Prolab (São Paulo, Brazil).

### 2.2. Bacterial Strains, Culture Media and Drugs for Biological Assays

We used *S. aureus* 1199B and K2068 strains to evaluate the antibacterial and inhibitory effect on NorA and MepA efflux pumps, which overexpressed the *NorA* and *MepA* genes, provided by Prof. Glenn Kaatz (Wayne State University School of Medicine) and Prof. Simon Gibbons (University College London), thereby encoding the NorA and MepA efflux pumps, respectively. The culture medium used was Brain Heart Infusion (BHI) (Sigma-Aldrich, St. Louis, MO, USA), prepared according to the manufacturer’s recommendations. The antibiotic used was norfloxacin (NOR) (Sigma-Aldrich, St. Louis, MO, USA) and the positive control was carbonyl cyanide *m*-chlorophenylhydrazone (CCCP) (Sigma-Aldrich, St. Louis, MO, USA). Norfloxacin was dissolved in dimethyl sulfoxide (DMSO) aqueous solution (5.1%, *v*/*v*) and 6CN-Ethyl dissolved in 2% methanol and 3% Tween 80 and water. The CCCP was dissolved in methanol/water (1:1, *v*/*v*). All solutions were prepared on the day of the experiments and used immediately, being kept at 20 °C during handling.

### 2.3. Component Selection

#### 2.3.1. Selection of Solid and Liquid Lipids

The lipids constituting the NLC matrix were selected based on the affinity of the drugs (6CN-Ethyl or norfloxacin) with the excipients. Compritol^®^ 888 ATO (Melting point-M.p: 74 °C), Precirol^®^ ATO (M.p: 57 °C), Gelucire^®^ 44/14 (M.p: 46 °C), Gelucire^®^ 43/01 (M.p: 44 °C), Gelucire^®^ 50/13 (M.p: 51 °C), and stearic acid (M.p: 69 °C), were evaluated for screening solid lipids. In each screening, 500 mg of the solid lipid was added to a 10 mL beaker and heated to 10 °C above its melting point, followed by the addition of 5 mg of the drug under magnetic stirring (C-MAG HS7, IKA^®^, São Paulo, Brazil) for 24 h. In turn, Labrafac^®^ Lipophile WL 1349, Transcutol^®^ HP, Capryol^®^ 90, Miglyol^®^ 812, and oleic acid were tested for liquid lipids. In each experiment, 500 μL of the lipid was added under magnetic stirring (300 rpm), followed by the addition of 15 mg of the drug. The samples were transferred to Petri dishes after 24 h and macroscopically evaluated for drug solubilization.

#### 2.3.2. Selection of Stabilizers

Pluronic^®^ F68, PVA, and Tween^®^ 80 (concentrations of 0.5%, 1%, and 3%) stabilizers were evaluated in this study based on the mean particle diameter, polydispersity index (PDI), and macroscopic appearance of the formulation. The formulations (F1–F9) were initially prepared using a 60:40 ratio between solid lipid and liquid lipid. The oil phase consisted of 60 mg of stearic acid and 40 mg of oleic acid, while the aqueous phase consisted of 10 mL of type II pure water (ORM-15, MARTE^®^, Santa Rita do Sapucaí, Brazil) and the stabilizer. The aqueous phase was added to the oil phase under magnetic stirring (C-MAG HS7, IKA^®^) for 5 min, followed by 3 min of sonication at 35% amplitude, using a 13 mm diameter titanium tip (Eco Sonics ultrasonic sonicator, 550 W, Indaiatuba, Brazil). Then, 10 mL of type II pure water (ORM-15, MARTE^®^) cooled to 5 °C were added to promote solidification of the nanoparticles.

#### 2.3.3. Selection of Solid–Liquid Lipid Ratio

The selection of the solid-to-liquid lipid ratio was performed according to the preparation method described previously (Section 2.3.2). First, three formulations (F10–F12) with different stearic acid:oleic acid ratios (60:40, 50:50, and 70:30) were tested in the oil phase. The ratio considered optimal was selected based on obtaining the smallest mean particle diameter and the lowest PDI.

#### 2.3.4. Selection of Sonication Amplitude and Time

A total of 15 formulations (F13–F27) were prepared based on the preparation method described in Section 2.3.2, using 1% PVA in the aqueous phase and a 60:40 stearic acid:oleic acid ratio in the lipid phase. The formulations were then sonicated with a time variation between 1 and 5 min and an amplitude between 30% and 70% in order to optimize the sonication parameters. After sonication, the lipid carriers were solidified by adding 10 mL of pure type II water cooled to 5 °C.

#### 2.3.5. Selection of Zwitterionic Surfactant Concentration

In this step, four formulations (F28–F31) containing different concentrations of lecithin (15, 25, 50, and 100 mg) incorporated into the oil phase were prepared. The formulations were developed based on the methodology described in Section 2.3.2, using the previously selected components: 1% PVA in the aqueous phase, a 60:40 lipid ratio (stearic acid:oleic acid), and sonication at 70% amplitude for 3 min. The samples were evaluated for mean particle diameter, PDI, and macroscopic appearance.

### 2.4. Preparation of Nanostructured Lipid Carriers Containing 6CN-Ethyl and Norfloxacin

NLCs containing 6CN-Ethyl and norfloxacin (NLC10NOR + 106CN) or without drugs (NLC-blank) were obtained by the hot emulsion sonication method. The oil phase was composed of stearic acid:oleic acid 60:40 and 25 mg of lecithin heated to 70 °C. For NLC10NOR + 106CN, after melting the lipids, 10 mg of 6CN-Ethyl and 10 mg of norfloxacin were added to the oil phase. The aqueous phase consisted of 1% (100 mg) of PVA and 10 mL of pure water type II. The aqueous phase was added to the oil phase under magnetic stirring (C-MAG HS7, IKA^®^) over 5 min, then subjected to sonication at 70% amplitude for 3 min (3 cycles of 1 min with a 30 s interval) in an ice bath. After the determined time, 10 mL of pure water type II cooled to 5 °C were added. Then, 2.5% trehalose was added to the formulation, and the samples were frozen for 24 h and lyophilized in a lyophilizer (Alpha 1-2 LDplus, CHRIST^®^, Osterode am Harz, Germany) for 24 h.

### 2.5. Characterization Study of Prepared Nanostructured Lipid Carriers

#### 2.5.1. Particle Size, Polydispersity Index and Zeta Potential

The hydrodynamic diameter and PDI of NLCs were obtained by dynamic light scattering (DLS) in a Zetasizer^®^ (Ultra Red, Malvern, Malvern, UK) device. The analyses were performed in replicates with a scattering angle of 173° at 25 °C. The zeta potential (ZP) was determined from the electrophoretic mobility under an electric field. The samples were diluted in a ratio of 10 μL of the sample to 990 μL of type II pure water (MARTE^®^ ORM-15, Prolab). All measurements were performed in triplicate, and all results are presented as mean and standard deviation.

#### 2.5.2. Determination of Encapsulation Efficiency

The encapsulation efficiency (EE%) of the drugs was determined using the indirect method. First, 500 μL of the NLC10NOR + 106CN formulation was transferred to a VIVASPIN 500 ultrafiltration tube (MWCO 10,000, Sartorius^®^, Göttingen, Germany) and centrifuged (5804r, Eppendorf^®^, Hamburg, Germany) at 14,000 rpm for 30 min. Then, 400 μL of the supernatant was collected, diluted in acetonitrile in a 10 mL flask, and subsequently analyzed by high-performance liquid chromatography (Shimadzu^®^ LC-20A Prominence model, Kyoto, Japan) at the detection wavelength of 278 nm for norfloxacin and 221 nm for 6CN-Ethyl employing the respective linear equations and regression coefficient (R^2^) (y = 39,963x − 17089, R^2^ = 0.9963) and (y = 34,237x + 1554.8, R^2^ = 0.9991). The corresponding calibration curves are presented in the Supplementary Materials (Figure S1). Next, EE% was determined in triplicate, and the value was calculated according to Equation (1):

(1) $$EE\left(\%\right)=\frac{Initial\;quantity-Free\;quantity}{Initial\;quantity}\times 100$$

The initial amount corresponds to the amount of norfloxacin or 6CN-Ethyl initially added to the formulation. The free quantity refers to the amount of norfloxacin or 6CN-Ethyl that remained free in the solution.

#### 2.5.3. Macroscopic Analysis of Nanostructured Lipid Carriers

Macroscopic analysis of NLCs was performed by direct visual inspection, observing color, homogeneity and possible presence of visible aggregates.

#### 2.5.4. Scanning Electron Microscopy (SEM)

The carriers were morphologically characterized using a scanning electron microscope (SEM) with a field emission gun (FEG) on MIRA3 (TESCAN) equipment. The NLC-blank and NLC10NOR + 106CN samples were previously resuspended in distilled water at a ratio of 1:100 (*v*/*v*). An aliquot was then deposited on aluminum supports, dried in a circulating air oven, and subsequently metallized with gold.

#### 2.5.5. Characterization by X-Ray Diffraction (XRD)

The diffraction patterns of the samples were determined by XRD using a diffractometer (Smartlab^®^, Rigaku, Akishima, Japan) equipped with a copper anode (λ = 1.5443 Å). The analyses were performed in 2θ with scanning of 5° and 60°, with a scanning speed of 5°/min and a step of 0.03°. The obtained diffractograms were analyzed in the Origin^®^ 8.5 (OriginLab Corporation, Northampton, MA, USA).

#### 2.5.6. Fourier Transform Infrared Spectroscopy (FTIR)

FTIR spectra were obtained using the attenuated total reflectance (ATR) technique using a spectrophotometer (IRSpirit, Shimadzu^®^, Kyoto, Japan). Analyses were performed in the spectral range of 4000 to 400 cm^−1^, with a resolution of 4 cm^−1^.

#### 2.5.7. Differential Scanning Calorimetry (DSC)

Thermal analysis of the samples was performed using a differential scanning calorimeter (DSC-60, Shimadzu^®^). Samples with an approximate mass of 2.5 ± 0.5 mg were deposited in hermetically sealed aluminum crucibles and subjected to a heating ramp from 25 °C to 300 °C, with a heating rate of 10 °C/min and a nitrogen flow rate of 50 mL/min. Thermal events and enthalpy were recorded and analyzed using the TA-60 software version 2.20 (Shimadzu^®^, Kyoto, Japan).

#### 2.5.8. In Vitro Drug Release Kinetics

The in vitro release kinetics of drugs encapsulated in nanostructured lipid carriers were evaluated using the dialysis membrane method, which were previously conditioned for 24 h in distilled water. The release medium consisted of a pH 7.4 sodium phosphate buffer solution containing 1% (*w*/*v*) Tween^®^ 80. The study was performed in triplicate for the NLC-blank and NLC10NOR + 106CN formulations, at a concentration of 0.5 mg mL^−1^ of norfloxacin and 0.5 mg mL^−1^ of 6CN-Ethyl, with the results expressed as a percentage of cumulative drug release. The dialysis membranes were loaded with 2 mL of the formulations and immersed in Erlenmeyer flasks containing 200 mL of the release medium, kept under constant agitation at 120 rpm, at 37 °C, in a shaker incubator (IKA KS 3000 i Control, Staufen im Breisgau, Germany).

Aliquots of 1 mL of the release medium were removed at predetermined time intervals (0.02, 0.08, 0.017, 0.25, 0.5, 1, 2, 3, 4, 5, 6, 7, 8, 9, 10, 12, 24, and 48 h), and an equivalent volume of sodium phosphate buffer was immediately replaced to maintain the sink condition. The samples collected were analyzed by HPLC, using the same parameters employed in the EE% determination. Subsequently, the in vitro drug release profile was analyzed using zero-order, first-order, Higuchi, Korsmeyer–Peppas, and Peppas–Sahlin kinetic models to determine the release mechanism. Model fitting was performed with the DDSolver add-in for Microsoft Excel, and the quality of fit was assessed based on correlation coefficient (R^2^) values [20].

### 2.6. Stability Study of Nanostructured Lipid Carriers

NLC-blank and NLC10NOR + 106CN formulation samples lyophilized with the addition of 2.5% trehalose, were stored in closed glass vials and kept refrigerated at 4 °C for 90 days. The formulations were subsequently evaluated for macroscopic aspects, mean particle diameter, PDI, and ZP at defined time intervals (1, 7, 14, 30, 60, and 90 days).

### 2.7. Evaluation of the Modulation of NLC Antibiotic Activity in S. aureus 1199B and S. aureus K2068 Strains by Minimum Inhibitory Concentration (MIC) Reduction

The study was performed using the broth microdilution method to verify the reduction in the Minimum Inhibitory Concentration (MIC) of NLC10NOR + 106CN against *S. Aureus 1199B* and *S. aureus K2068* strains, as these strains overexpress the NorA and MepA efflux pumps, respectively. Bacterial inocula of both strains were prepared in sterile saline solution according to the 0.5 McFarland scale, corresponding to 1.5 × 10^8^ Colony Forming Units (CFUs). Six groups were tested: free 6CN-Ethyl, norfloxacin (negative control), physical mixture 6CN-Ethyl + norfloxacin (a solution prepared with 500 μg/mL of 6CN-Ethyl and norfloxacin), CCCP (carbon cyanide m-chlorophenylhydrazone) + norfloxacin (positive control), NLC-blank, and NLC10NOR + 106CN, which consisted of the test sample containing 500 μg/mL of 6CN-Ethyl and norfloxacin in the nanocarrier; all assays were performed in triplicate. Dilution media were prepared in microtubes containing 900 μL of Brain Heart Infusion (BHI) medium and 100 μL of the bacterial inoculum. Next, 100 μL of the tube contents were transferred to a 96-well microdilution plate. Serial microdilution (1:1) was subsequently conducted with 100 µL of the substances from each group tested. Microdilution was performed up to the penultimate well, leaving the last well as a growth control. The plates were incubated in a bacteriological incubator for 24 h at 37 °C. The experiments were performed in triplicate. The reading was performed by adding 20 µL of resazurin (7-hydroxy-3H-phenoxazine-3-one 10-oxide), observing the color change in the medium in each well. Blue coloration indicated the absence of bacterial growth, while a color change to red indicated bacterial growth.

### 2.8. Statistical Analysis

The results were expressed as mean ± standard deviation, and the experiments were performed in triplicate. Data normality was assessed using the Shapiro–Wilk test. Comparisons between experimental groups were performed using two-way analysis of variance (two-way ANOVA), followed by Tukey’s post hoc test. Differences were considered statistically significant at *p* < 0.05.

The assays for the analysis of efflux pump inhibition by MIC were performed in triplicate, and the results were compared using one-way ANOVA, followed by Dunnett’s post hoc test. The results were expressed as geometric mean ± standard error of the mean (SEM), with values considered significant at *p* < 0.05. The GraphPad Prism^®^ 5.0 software was used for statistical analyses.

## 3. Results and Discussion

### 3.1. Determination of Components for the Preparation of Nanostructured Lipid Carriers

#### 3.1.1. Selection of Solid and Liquid Lipids

The study to select the most appropriate solid lipid was conducted over 24 h, heating to a temperature 10 °C above the melting point of each lipid tested. The evaluation was based on the solubility of norfloxacin and 6CN-Ethyl. The 6CN-Ethyl derivative showed rapid solubilization in stearic acid, followed by Gelucire^®^ 43/01, while norfloxacin showed complete solubilization in stearic acid. Thus, stearic acid was chosen as the solid lipid for subsequent testing because allow the drug solubilization without the need for high temperatures during preparation, due to its lipid matrix offering greater flexibility for incorporating active ingredients, and it is a widely used excipient in food and pharmaceutical formulations [21].

The liquid lipid selection subsequently demonstrated that 6CN-Ethyl presented complete solubilization in all lipids tested. However, oleic acid stood out in the first hours of the experiment for initiating the solubilization process more quickly than the others. In contrast, norfloxacin was completely solubilized only in oleic acid.

Oleic acid was selected as the liquid lipid in the formulation because it was able to fully solubilize both molecules and is also described in the literature as an enhancer of the physical stability of colloidal systems, favoring NLC efficacy [22]. The selection of solid and liquid lipids directly influences the internal structure, drug loading capacity, release behavior of the NLCs, and overall therapeutic efficacy. In addition, the lipids should solubilize the active compounds to ensure uniform distribution and prevent linkage and precipitation of the actives. The choice of lipids influences the long-term storage stability, the encapsulation efficiency, and particle size [23,24].

#### 3.1.2. Selection of Stabilizers

After selecting the appropriate lipids for the formulation, nine formulations (F1–F9) were prepared, varying the type and concentration of stabilizers (see Table 1). Particle diameters ranged from 268 to 1283 nm, with PDI values between 0.171 and 0.77. The formulation with the smallest particle size, the lowest PDI value, and the absence of lipid droplets or phase separation was chosen to select the stabilizer and its concentration.

All stabilizers could produce NLC at the nanoscale, making it impossible to directly compare the type of stabilizer used and its concentration with particle size. Regarding the influence of stabilizers on system homogeneity, formulations with Pluronic and PVA showed a reduction in PDI value as the concentration increased. However, this difference was only statistically significant when compared to a concentration of 0.5%. The decrease in PDI observed with increasing concentrations of Pluronic^®^ and PVA can be attributed to improved surface coverage of the lipid carriers, which reduces interfacial tension and enhances steric stabilization during particle formation, resulting in a narrower particle size distribution. However, despite the reduction in PDI values, this improvement was not reflected in long-term physical stability. Moreover, most stabilizers form formulations with the presence of oil droplets or aggregates.

Formulations containing Tween^®^ showed a concentration-dependent effect, contributing to an increase in the mean diameter and PDI parameters. In contrast to the formulations of 0.5% and 1.0% of Tween^®^, the formulation with 3.0% concentration resulted in a highly significant increase in these parameters, as well as phase separation of the formulation, indicating that excess of Tween^®^ negatively affected the stability of the system.

Only the F5 and F6 formulations presented acceptable macroscopic properties, mean diameter, and PDI. Therefore, PVA at a concentration of 1% was selected as the stabilizer, as it best met the selection criteria, considering the smallest particle diameter, PDI, and macroscopic stability. PVA is commonly used as an emulsifier and stabilizer in the preparation of NLC, preventing aggregation and ensuring uniform particle size distribution. The stability promoted by the addition of PVA was previously reported by de Ghani et al. (2021), in which NLC containing *Ficus deltoidea* extracts were developed formulated from stearic acid, oleic acid, and PVA, and reported improved dispersion stability maintenance over shelf life [25]. Furthermore, PVA is a biocompatible, biodegradable, flexible, and low-toxicity polymer, constituting characteristics which make it an excellent candidate as a stabilizer for NLC in healthcare applications [26].

#### 3.1.3. Selection of Solid–Liquid Lipid Ratio

Three formulations (F10–F12) were prepared to select the optimal stearic acid:oleic acid ratio. NLCs are composed of a mixture of solid and liquid lipids, which form a less ordered lipid matrix, enhancing drug loading capacity and stability. The choice of the proportion of lipids affects the particle size, entrapment efficiency, and release kinetics of the encapsulated drug, making it a critical factor in the design of effective NLC systems.

The increase in the oleic acid proportion led to a change in particle size, with the smallest size obtained with 40% oleic acid (336 nm). It was also possible to observe a reduction in PDI with the increase in the oleic acid proportion in the formulation (Table 2). Similar results were reported by Latifah et al. (2024) [27], who used the same lipid ratio (60:40) in formulations containing diclofenac. An increase in the proportion of oleic acid promoted a reduction in particle size, an effect that can be attributed to the decrease in the viscosity of the lipid phase, favoring the formation of smaller carriers [27]. Nevertheless, an increase in the liquid lipid volume may impair the efficacy of the stabilizer, leading to an increase in surface tension and to forming larger particles [28]. These data indicate that the suspension stability is not only related to the increase in the amount of liquid lipid in the matrix, but also to the adequate proportion of solid lipid, which gives greater stability to the system. This proportion, associated with the PVA concentration, reduced the viscosity and surface tension of the lipid droplets, resulting in smaller particles [29]. In view of the above, the formulation containing the lipid ratio of 60:40 was selected for subsequent tests.

#### 3.1.4. Selection of Sonication Amplitude and Time

The formulations were prepared by varying the amplitude and sonication time. The results presented in Table 3 demonstrate that prolonged sonication significantly reduces particle diameters. However, the average particle diameter for amplitudes of 50 and 70% increased after 3 min of sonication. As reported by Ajiboye et al. (2021) [30], prolonged sonication can cause the opposite phenomenon, leading to increased particle size. This effect can be attributed to excessive acoustic cavitation, which generates intense microjets and shock waves, promoting particle collision, coalescence, and re-agglomeration of previously formed nanoparticles. In addition, prolonged sonication may induce local temperature increases and high shear stress, destabilizing the lipid–surfactant interface and reducing the effectiveness of steric stabilization. NLC subjected to physical stress for long periods can produce unstable colloidal systems and even promote lipid oxidation, making it essential to optimize the ideal sonication time [30].

Amplitude refers to the intensity of the ultrasonic waves applied during sonication. Higher amplitudes generally provide more energy, leading to smaller particle sizes due to increased cavitation effects [31]. Nevertheless, a stabilization in nanoparticle size or even an increase in average diameter is observed after adding sufficient energy to the system.

These results indicate that higher amplitudes, combined with moderate sonication times, are effective in obtaining nanoparticles with smaller diameters, good homogeneity, and stability, avoiding excessive heating of the system [32]. Considering these factors, the selected formulation was F25, subjected to sonication at 70% amplitude for 3 min, which showed an average diameter of 184.5 nm and a PDI of 0.29. No agglomeration or phase separation were observed, indicating the physical stability of the system.

#### 3.1.5. Selection of Zwitterionic Surfactant Concentration

Different lecithin concentrations (Table 4) were added to the formulation to mitigate issues related to colloidal stability and shelf life. Lecithin can form an electrical double layer on the surface of NLCs due to its ionic properties, increasing their surface charge. Mixing an ionic surfactant with a non-ionic stabilizer, such as PVA, can form more stable NLCs [28].

It seems that the lectin concentration plays an important role in stabilizing the NLC. It was observed that the formulation containing 25 mg of lecithin presented the best results, with a mean diameter of 233.4 nm and a PDI of 0.25, indicating that lecithin at this concentration more effectively contributes to the system stability [33,34]. On the other hand, higher concentrations resulted in an increase in particle diameter and PDI, suggesting possible saturation of the interface. Although the one-way ANOVA test followed by Tukey’s test did not indicate statistically significant differences in particle diameter between the groups, a significant difference in PDI was observed between the formulations with 25 mg and 100 mg of lecithin (*p* < 0.05). These results agree with Schubert et al. (2006), who reported a concentration-dependent effect of lecithin on lipid nanoparticle size with an initial reduction in diameter, followed by stabilization and subsequent increase, associated with the accumulation of surfactant at the interface and the consequent instability of the system at high concentrations [35]. The F29 formulation was established as NLC-blank.

### 3.2. Characterization of Nanostructured Lipid Carriers

#### 3.2.1. Determination of Encapsulation Efficiency

After NLC optimization, 10 mg of 6CN-Ethyl and 10 mg of NOR were incorporated into the system (NLC10NOR + 106CN). The EE% of NLC10NOR + 106CN was 99.50% for 6CN-Ethyl and 90.91% for norfloxacin, demonstrating the ability of NLCs to encapsulate both molecules. According to Haider et al. (2020), EE% values greater than 60% indicate an efficient encapsulation process associated with adequate drug incorporation into the lipid matrix [36]. The high encapsulation efficiency can be attributed to the liquid lipid, as it limits recrystallization of the lipid matrix, favoring formation of amorphous or imperfect crystalline structures, which facilitate drug incorporation. It also provides greater structural stability to the system, reducing drug losses during preparation and storage [37].

#### 3.2.2. X-Ray Diffraction (XRD)

**Figure 2:**
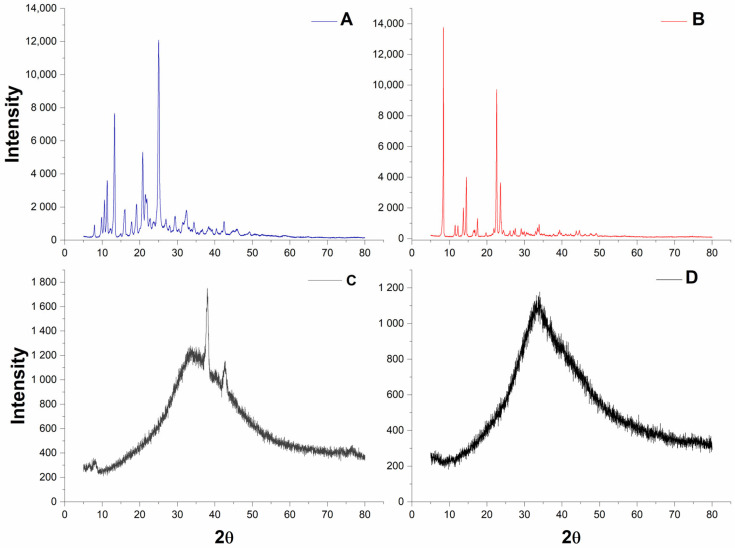
Diffractogram of 6CN-Ethyl, norfloxacin, NLC, NLC10NOR + 106CN. (**A**)—Norfloxacin; (**B**)—6CN-Ethyl; (**C**)—NLB-blank; (**D**)—NLC10NOR + 106CN. NLC-blank—Drug-free nanostructured lipid carrier. NLC10NOR + 106CN—Nanostructured lipid carrier with 10 mg of norfloxacin and 10 mg of 6CN-Ethyl.

The diffractograms of 6CN-Ethyl and norfloxacin exhibited structural ordering along the 2θ angles, suggesting a crystalline structure (Figure 2). On the other hand, the diffractograms of NLC-blank and NLC10NOR + 106CN (Figure 2) revealed the appearance of an amorphous halo, characteristic of disordered materials, such as NLCs, which extends widely from approximately 10° to 60° (2θ), caused by the presence of liquid lipid in the formulation. This characteristic corroborates the high values found for EE%, since the disordered matrix enables greater drug incorporation and indicates that the drugs were completely solubilized in the lipids of the amorphous matrix, without the presence of drug-related peaks. Similar findings were reported in a study by Idris et al. (2023), in which an NLC containing docetaxel was developed, and XRD analysis revealed the appearance of an amorphous fraction when compared to the crystalline signals of the free components [38,39].

#### 3.2.3. Scanning Electron Microscopy (SEM)

**Figure 3:**
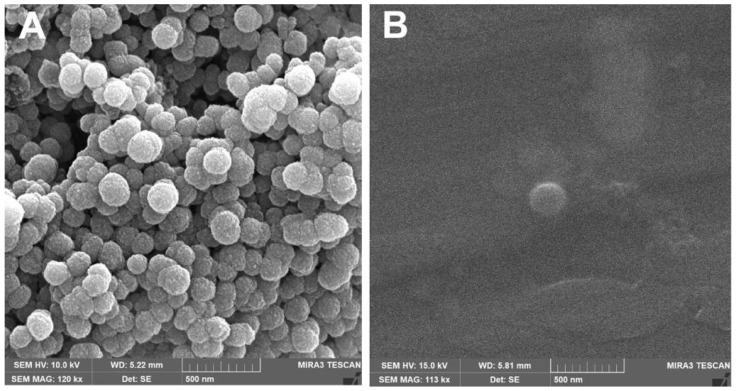
Morphological image by scanning electron microscopy (SEM) of NLC-blank and NLC10NOR + 106CN. (**A**)—NLC-blank; (**B**)—NLC10NOR + 106CN. NLC-blank—Drug-free nanostructured lipid carrier. NLC10NOR + 106CN—Nanostructured lipid carrier with 10 mg of norfloxacin and 10 mg of 6CN-Ethyl.

The morphological analyses of the nanostructured lipid carriers (NLCs) obtained by SEM-FEG are shown in Figure 3. Both NLC-blank and NLC10NOR + 106CN exhibited particles with a predominantly spherical morphology, and the observed size was within the nanometric range. The spherical morphology of the NLCs can be attributed to homogenization followed by the cooling process, which favors the formation of lipid droplets with lower surface energy and a spherical geometry. Similar findings were reported by Ângelo et al. (2020), who described nanostructured lipid carriers with spherical morphology and an average diameter in the nanometric scale [40].

Additionally, the microscopy images show that some particles appear in close proximity to each other, which may be related to the lipid nature of the carrier and the drying process during sample preparation for SEM analysis, rather than reflecting the behavior of the system in the dispersed state.

#### 3.2.4. Fourier Transform Infrared Spectroscopy (FTIR)

**Figure 4:**
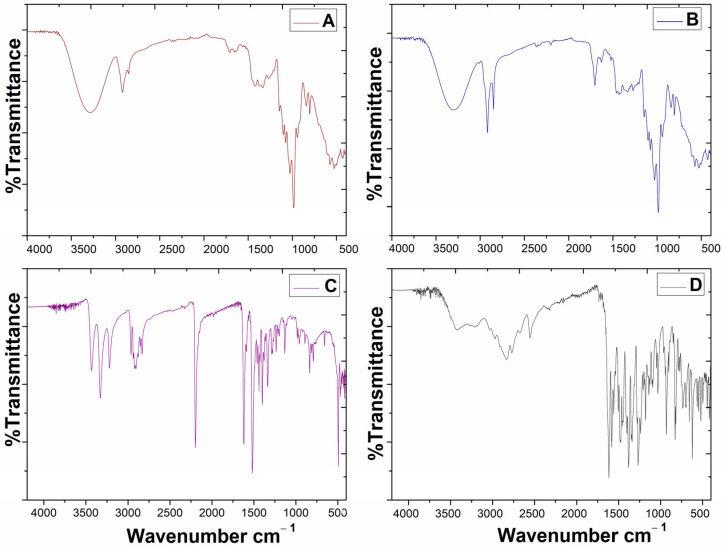
Infrared spectra of 6CN-Ethyl, norfloxacin, NLC, NLC10NOR + 106CN. (**A**)—NLC-blank; (**B**)—NLC10NOR + 106CN; (**C**)—6CN-Ethyl; (**D**)—Norfloxacin. NLC-blank—Drug-free nanostructured lipid carrier. NLC10NOR + 106CN—Nanostructured lipid carrier with 10 mg of norfloxacin and 10 mg of 6CN-Ethyl.

The FTIR spectra of 6CN-Ethyl, norfloxacin, NLC-blank and NLC10NOR + 106CN are shown in Figure 4. The spectrum of the thiophene derivative 6CN-Ethyl exhibited peaks at 2196 cm^−1^ (-CN), 1518 and 1619 cm^−1^ (C=C), 3330 and 3433 cm^−1^ (-NH_2_) and 2905 cm^−1^ (C-H), confirming its chemical structure. The FTIR spectrum of norfloxacin showed characteristic displacements of the molecule, a broad band between 3500 and 2500 cm^−1^ (O–H), 2552 cm^−1^ (N–H of primary amine), 1724 cm^−1^ (C=O of carboxylic acid), 1629 cm^−1^ (C=O of ketone), 1581 cm^−1^ (carbonyl group of the quinolone system) and 941 and 931 cm^−1^ (out-of-plane vibrations of the aromatic C–H of the quinoline ring), confirming its identity [41].

Finally, the spectra of the lipid carriers showed characteristic bands of the formulation components. The spectra of NLC-blank and NLC10NOR + 106CN exhibited a broad band between 3400 and 3200 cm^−1^ (O–H of PVA, trehalose, and lecithin), bands at 2920 and 2850 cm^−1^ (C–H of the aliphatic chains of oleic acid, stearic acid, and lecithin), a peak at 1700 cm^−1^ (C=O of fatty acids), and a peak at 1140 cm^−1^ (C–O–C ether bond) of PVA. However, no new peaks were observed, indicating that there was no chemical interaction between the drugs and the NLC components, which is important to ensure the system’s stability and efficacy [42,43].

#### 3.2.5. Differential Scanning Calorimetry

**Figure 5:**
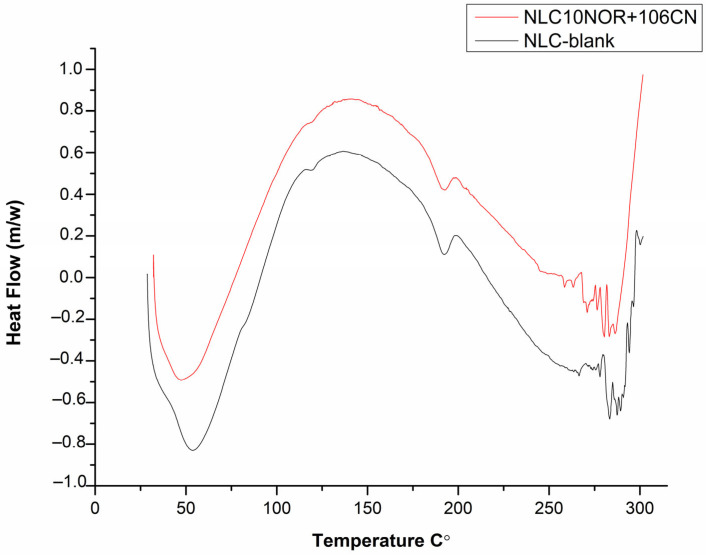
DSC thermograms of 6CN-Ethyl, norfloxacin, NLC, NLC10NOR + 106CN. NLC-blank—Drug-free nanostructured lipid carrier. NLC10NOR + 106CN—Nanostructured lipid carrier with 10 mg of norfloxacin and 10 mg of 6CN-Ethyl.

A thermal DSC analysis of the NLC-blank and NLC10NOR + 106CN samples is presented in Figure 5. The results revealed an endothermic peak around 50 °C, attributed to the melting point of stearic acid. According to Ribeiro et al. (2012), the shift in relation to the typical value (~70 °C) suggests a reduction in the crystallinity of the lipid matrix, influenced by the presence of oleic acid [44]. This behavior indicates disorganization of the lipid structure and favoring formation of a more amorphous matrix, as evidenced in the XRD analyses. In addition, a broad signal can be observed between 50 and 180 °C, which can be attributed to the PVA, lecithin, and trehalose present in the formulation. Trehalose dihydrate generally exhibits an endothermic dehydration peak between 95 and 100 °C. In turn, lecithin exhibits phase transitions between 120 and 150 °C, while PVA exhibits the endothermic melting or decomposition event above 180 °C. The absence of distinct thermal signals from these excipients reinforces the hypothesis of physicochemical interactions and the formation of a molecularly homogeneous matrix [45,46].

In the formulation containing norfloxacin and 6CN-Ethyl, a thermal profile similar to that of the system without the drugs was observed, with subtle changes in the signal shape and intensity, suggesting an additional reduction in crystallinity due to the interaction of the drugs with the matrix components [47]. Absence of the endothermic peak characteristic of norfloxacin, whose melting point is reported in the literature at around 220 °C, as well as the lack of a defined signal in the range of 145–151 °C, attributed to the 6CN group of the thiophene derivative, suggest efficient encapsulation of the active ingredients [48,49,50]. These results are in agreement with the findings of Mello and Ricci-Júnior, [47] who observed complete disappearance of the drug’s melting peak in the DSC thermograms when encapsulating naproxen in polymeric polycaprolactone nanoparticles, evidencing the amorphization of the compound and its interaction with the matrix polymer.

#### 3.2.6. In Vitro Drug-Release Kinetics

**Figure 6:**
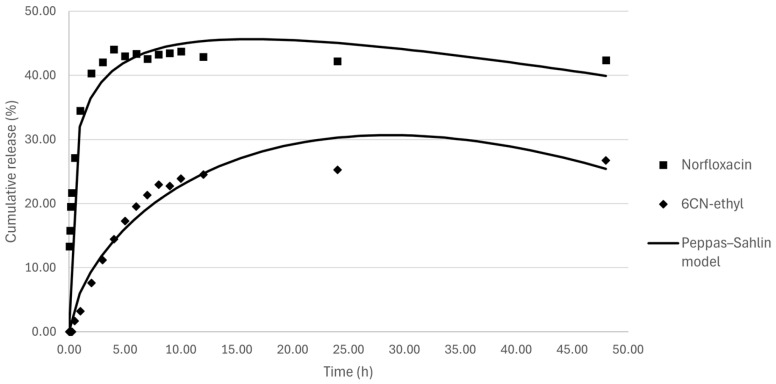
Evaluation of the in vitro release profile of the norfloxacin and 6CN-ethyl in NLC10NOR + 106CN. NLC10NOR + 106CN—Nanostructured lipid carrier with 10 mg of norfloxacin and 10 mg of 6CN-Ethyl.

The in vitro release profiles, expressed as the percentage of cumulative drug release, were monitored over a 48 h period for both NOR and 6CN-Ethyl from the NLC using the dialysis membrane method. As illustrated in Figure 6, the release behaviors of the two co-encapsulated drugs were distinct. NOR exhibited a rapid initial release, reaching approximately 40% cumulative release within the first few hours, followed by a sustained release phase over the remaining period, eventually reaching over 42% by 48 h. In contrast, 6CN-ethyl demonstrated a slower and more gradual release, with cumulative release steadily increasing over time but at a lower overall percentage compared to norfloxacin within the same timeframe, reaching around 26% by 48 h. The initial burst release observed, particularly for NOR, is attributed to the presence of drug adsorbed on the NLC surface or drug enriched in the liquid lipid domains (oleic acid). The initial rapid release ensures a quick onset of action, while the subsequent sustained phase maintains therapeutic concentration over time [51]. The different cumulative release for NOR and 6CN-ethyl are likely due to the physicochemical properties of the two drugs, such as their partition coefficients, molecular size and specific interactions with the NLC components (solid lipid, liquid lipid, and surfactants).

To elucidate the drug release mechanisms, the experimental data were fitted to Zero-order, First-order, Higuchi, Korsmeyer–Peppas, and Peppas–Sahlin mathematical models. The coefficient of determination (R^2^) was used to assess the quality of fit [52].

The Peppas–Sahlin model provided the best fit for both drugs, with R^2^ values of 0.9597 for norfloxacin and 0.9603 for 6CN-Ethyl. These results suggest that the release of both drugs from the NLCs is best described by the Peppas–Sahlin model, which are often indicative of complex release mechanisms involving diffusion and erosion processes, typical for lipid-based matrices [53].

The Peppas–Sahlin model is described by the equation: M(t)/M_∞_ = k_1_·t^m^ + k_2_·t^2m^, where M(t) and M_∞_ are the amount of drug released at time t and the total amount released at equilibrium k_1_ and k_2_ are constants related to diffusion and relaxation [53,54].

The NOR release profile is characterized by an exponent, m = 0.280, indicative of Fickian diffusion. This suggests that the release of NOR is primarily controlled by the concentration gradient across the NLC matrix, with minimal contribution from the structural relaxation/erosion of the lipid carrier [53]. The high positive value of k_1_ (41.898 h^−0.280^) against the negative k_2_ value (−9.586 h^−0.560^) supports the dominance of the diffusion component. The rapid diffusion rate (k_1_) correlates with the burst release observed in Figure 6. Previous studies on norfloxacin-loaded lipid systems, such as nanoemulsions and lipospheres, reported enhanced solubility and controlled release, with diffusion-driven kinetics dominating the early phase [55,56]. Our findings corroborate these reports, but the strong fit to the Peppas–Sahlin model suggests that matrix relaxation/degradation also plays a role in NLC systems, likely due to the complex lipid matrix.

The 6CN-Ethyl release profile is characterized by a significantly higher exponent m of 0.670, indicating an anomalous transport (non-Fickian). This confirms that the release of 6CN-ethyl is governed by a combination of drug diffusion through the lipid matrix and structural changes in the NLC. The positive and dominant k_1_ (6.481 h^−0.670^) compared to the negative k_2_ (−0.342 h^−1340^) suggests that diffusion is the primary rate-limiting step, with the relaxation component being negligible or poorly modeled by the equation [53].

Ortiz et al. (2021) demonstrated that NLCs often exhibit a complex release, with complex based models providing superior fitting compared to classical zero- or first-order kinetics [57]. Similarly, Porbaha et al. (2024) highlighted that lipid nanoparticles frequently show a release mechanism that involves a combination of diffusion, swelling, and erosion, that fits with an anomalous transport, requiring dual-mechanism models for accurate description [58]. Our data aligns with these observations, reinforcing the importance of advanced kinetic modeling in NLC research.

### 3.3. Stability Study of Nanostructured Lipid Carriers

The lyophilized NLC-blank and NLC10NOR + 106CN were stored under refrigeration for 90 days and analyzed for particle diameter, PDI, and zeta potential. The results are presented in Table 5.

Particle diameter analysis revealed that the NLC-blank had an average size of 196 nm and 232 nm at D1 and D90, respectively. The average size fluctuated by 36 nanometers throughout the study. This variation can be considered acceptable and corroborates the stability of the NLC-blank during this analysis period. The drug-containing carrier had a size close to 240 nm in the first 60 days of the study, indicating that this system demonstrated good stability during this period. However, on day 90, the NLC10NOR + 106CN showed a significant increase in its average size (349 nm), indicating system instability.

The drug-containing nanoparticles had a slightly larger average size than the blank nanoparticles. The increase in particle diameter after drug encapsulation has been reported in previous studies and may be attributed to the characteristic structure of NLCs, in which the addition of the active ingredient promotes structural reorganization with the lipid matrix, resulting in an increase in particle volume [36,59].

Analysis of PDI values indicates an increase after drug encapsulation; however, the ANOVA test revealed no significant differences over the 90 days and when comparing NLC-blank and NLC10NOR + 106CN. In addition, PDI values were <0.4, a value reported in the literature as representing populations with good homogeneity, ensuring system stability and safety [60].

NLC-blank presented ZP between −23 and −28 mV, values considered adequate for nanoparticle stability, except for D1 (−17.52). Initial variations followed by stabilization indicate system equilibrium, which is a relevant factor for increasing shelf life and maintaining system integrity during storage [61]. On the other hand, NLC10NOR + 106CN presented ZP lower than −30 mV in the first 30 days of analysis, indicating electrostatic stability of the system, conferring greater repulsion between particles and minimizing particle aggregation [62,63]. However, a significant increase in negative charge was observed from day 60 onwards compared to the initial days of storage, suggesting the onset of colloidal instability processes and possible nanoparticle aggregation. These data are corroborated by the increase in average particle size in D90 and highlight the importance of ZP in ensuring NLC stability.

### 3.4. Evaluation of the Modulation of the Nanostructured Lipid Carrier Antibiotic Activity in S. aureus 1199B and S. aureus K2068 Strains

**Figure 7:**
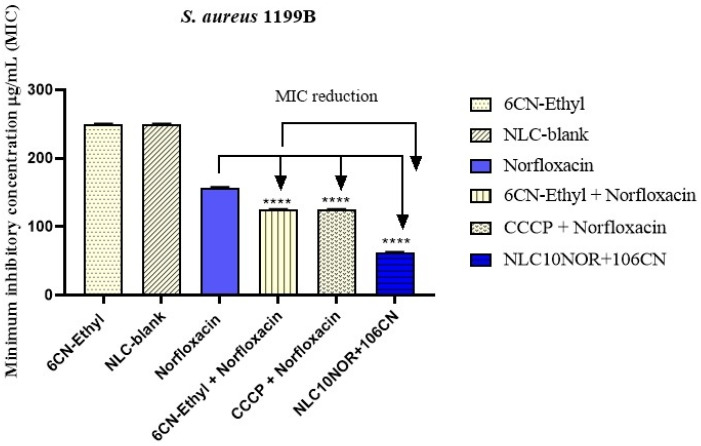
Minimum inhibitory concentration (MIC) of *S. aureus* 1199B strain. NLC-blank—Drug-free nanostructured lipid carrier. NLC10NOR + 106CN—Nanostructured lipid carrier with 10 mg of norfloxacin and 10 mg of 6CN-Ethyl. CCCP- carbonyl cyanide *m*-chlorophenyl hydrazone. **** *p* < 0.0001. The assays were performed in triplicate, and the results were compared using one-way ANOVA, followed by Dunnett post hoc. The results were expressed as geometric mean ± standard error of the mean (SEM), with values considered significant when *p* < 0.05.

**Figure 8:**
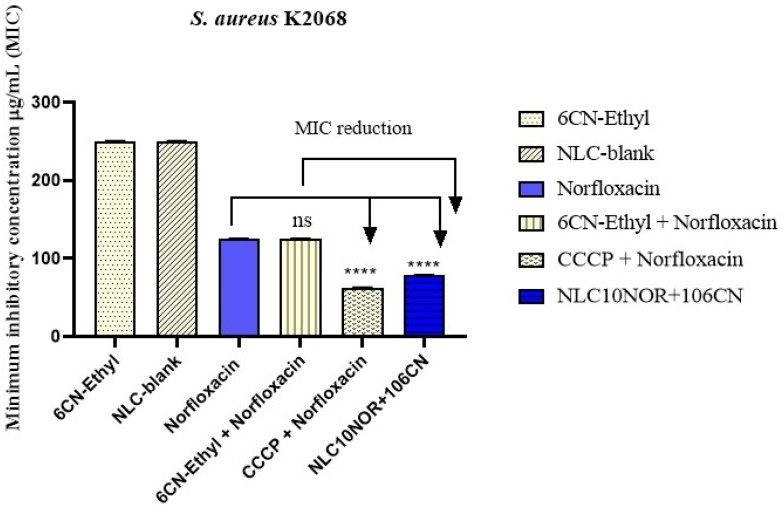
Minimum inhibitory concentration (MIC) of *S. aureus* K2068 strain. NLC-blank—Drug-free nanostructured lipid carrier. NLC10NOR + 106CN—Nanostructured lipid carrier with 10 mg of norfloxacin and 10 mg of 6CN-Ethyl. ns—no significancy. CCCP—carbonyl cyanide *m*-chlorophenyl hydrazone. **** *p* < 0.0001. The assays were performed in triplicate, and the results were compared using one-way ANOVA, followed by Dunnett post hoc. The results were expressed as geometric mean ± standard error of the mean (SEM), with values considered significant when *p* < 0.05.

The effects on modulation of the antibiotic activity of norfloxacin in combination with the 6CN-Ethyl efflux pump inhibitor incorporated into the NLC were evaluated. The MIC determination assay was used for norfloxacin, 6CN-Ethyl, the physical mixtures norfloxacin + 6CN-Ethyl and norfloxacin + CCCP, the NLC-blank and the NLC10NOR + 106CN against each of the resistant *S. aureus* strains. The results can be seen in Figure 7 and Figure 8.

In Figure 5, compounds, physical mixtures and NLCs were evaluated against the *S. aureus* 1199B strain, which overexpresses the NorA efflux pump.

It was observed that neither 6CN-Ethyl nor NLC-blank exhibited the ability to inhibit the growth of the *S. aureus 1199B* strain (MIC > 200 μg/mL ± 2), demonstrating that they are free of antibiotic activity. Similarly, and as expected, norfloxacin alone also did not show antibiotic activity against the *S. aureus 1199B* strain (MIC = 157.5 μg/mL ± 0.9) because this strain is resistant to fluoroquinolones.

The physical mixtures norfloxacin + CCCP and norfloxacin + 6CN-Ethyl were able to equipotently reduce resistance to norfloxacin by *S. aureus 1199B*, resulting in MIC values equal to 125 ± 2 μg/mL. This value has statistical difference (*p* < 0.0001) when compared to the MIC value of norfloxacin alone, showing that the 2-amino-thiophene derivative 6CN-Ethyl has NorA efflux pump inhibitory activity equipotent to the CCCP against the *S. aureus 1199B* strain.

The NLC10NOR + 106CN resulted in a MIC value = 62.5 ± 0.9 μg/mL, being two times lower than the MIC of the physical mixture norfloxacin + 6CN-Ethyl, and two-and-a-half times lower than the MIC of norfloxacin (values with statistically different (*p* < 0.0001)), thus indicating that the incorporation of norfloxacin and 6CN-Ethyl in the NLC was more efficient in modulating antibiotic activity and reducing resistance to norfloxacin by the *S. aureus 1199B* strain than the physical mixture.

Compounds, physical mixtures and NLCs were evaluated against the *S. aureus K2068* strain in Figure 8, which overexpresses the MepA efflux pump.

Similarly to what was observed against the *S. aureus 1199B* strain, neither 6CN-Ethyl nor NLC-blank exhibited the ability to inhibit the growth of the *S. aureus K2068* strain (MIC > 200 ± 2 μg/mL), demonstrating that they are free of antibiotic activity. Norfloxacin alone also did not show antibiotic activity against the *S. aureus K2068* strain (MIC = 125 ± 1 μg/mL), since this strain also shows resistance to fluoroquinolones due to overexpression of the gene encoding the efflux pump MepA.

Unlike what was observed against the *S. aureus 1199B* strain, the physical mixtures norfloxacin + CCCP and norfloxacin + 6CN-Ethyl present distinct activity profiles against the *S. aureus K2068* strain. The physical mixture norfloxacin + CCCP promoted a two-fold reduction in MIC (MIC = 62.5 ± 0.9 μg/mL), demonstrating the efficiency of CCCP in also inhibiting the MepA efflux pump, helping to reduce the strain’s resistance to norfloxacin. The physical mixture norfloxacin + 6CN-Ethyl presented a MIC value equivalent to that of norfloxacin alone (MIC = 125 ± 2 μg/mL), indicating the inability of the 6CN-Ethyl compound to act as an inhibitor of the MepA pump when physically combined.

According to Kumawat et al. (2023) [64] and Maldonado et al. (2023) [65], the MATE family pumps are H^+^/Na^+^ antiport systems that extrude cationic substrates. 6CN-Ethyl in free form and in the physical mixture with norfloxacin possibly does not effectively interact with the MepA binding site because it is a weak base. On the other hand, according to Pasqua et al. (2019) [66], the NorA pump has structural affinity with several substrates such as sugars, drugs, amino acids, and metabolites, which may explain the better inhibition activity observed in the physical mixture of 6CN-Ethyl with norfloxacin against the *S. aureus 1199B* strain.

In contrast, incorporating norfloxacin and 6CN-Ethyl into NLC resulted in modulating the antibiotic activity and reduced norfloxacin resistance in the *S. aureus K2068* strain, resulting in a MIC = 78.41 ± 0.87 μg/mL. This value is 38% lower, as well as statistically different (*p* < 0.0001) than the MIC values observed with norfloxacin alone, and with the physical mixture norfloxacin + 6CN-Ethyl.

However, despite the need to develop new strategies to overcome antimicrobial resistance, studies involving co-encapsulation of fluoroquinolones and efflux pump inhibitors in lipid nanostructured systems are still scarce in the literature. However, the improvement of fluoroquinole activity after encapsulation in lipid nanoparticles has already been reported in the literature, as in the study by Dlamini et al. (2025) [67], where lipid carriers containing eugenol and D-α-tocopherol were developed for encapsulating ciprofloxacin (CIP-NLCs). It was observed that encapsulation in CIP-NLCs promoted a 2-fold reduction in the MIC compared to free ciprofloxacin against MRSA, *Staphylococcus aureus*, *Escherichia coli* and *Pseudomonas aeruginosa* [67]. Similar results were observed in research with other antibiotics. For example, Andrade et al. (2023) reported that encapsulating vancomycin in solid lipid nanoparticles promoted a 22-fold reduction compared to the free drug against MRSA strains [68]. Furthermore, it was reported by Dong et al. (2021) that the presence of stearic acid as a solid lipid in solid nanoparticles containing norfloxacin promoted a significant increase in its bioavailability and the antibacterial activity of the antibiotic against *Staphylococcus aureus* [69].

Therefore, the present study stands out as a novel, innovative, and promising approach for modulating antimicrobial resistance against multidrug-resistant *Staphylococcus aureus* strains overexpressing NorA and MepA efflux pumps through development of nanostructured lipid carriers coencapsulating norfloxacin and an efflux pump inhibitor (2-amino-thiophene derivative 6CN-Ethyl). These results demonstrate that encapsulation enhances the activity of the compounds when compared to the action of norfloxacin and 6CN-Ethyl alone.

## 4. Conclusions

This study highlights the potential of nanostructured lipid carriers (NLCs) as a promising carrier system in pharmaceutical sciences, particularly in the context of antimicrobial resistance. NLCs were successfully prepared via hot emulsion sonication and optimized for co-encapsulation of norfloxacin and the 6CN-Ethyl efflux pump inhibitor. The NLC10NOR + 106CN formulation demonstrated physicochemical stability for up to 60 days, with high encapsulation efficiency (>90%) and a complex profile of release for both active compounds. Characterization by FTIR, DSC, SEM and XRD confirmed effective encapsulation, as evidenced by the disappearance of characteristic peaks and the emergence of amorphous structures. Antibacterial assays revealed that NLC10NOR + 106CN significantly reduced the minimum inhibitory concentration (MIC) against *Staphylococcus aureus K2068* and *1199B* strains, outperforming the physical mixture of norfloxacin and 6CN-Ethyl. Notably, the formulation showed modulation of 6CN-Ethyl activity in the *S. Aureus 1199B* strain, suggesting inhibition of efflux pump mechanisms. These findings support an innovative therapeutic strategy based on the simultaneous encapsulation of an antibiotic and an efflux pump inhibitor in NLCs, a combination not previously reported in the literature. This approach effectively restores bacterial sensitivity to norfloxacin and may contribute to prolonging its clinical efficacy. Therefore, the proposed system offers a novel and relevant perspective for addressing multidrug-resistant bacterial infections.

## Figures and Tables

**Table 1:** Particle diameter and polydispersity index of formulation with different stabilizers.

Formulations	Stabilizers	Particle Diameter	Polydispersity Index	Macroscopic Aspects
F1	Pluronic^®^ 0.5%	268 ^a^ (±6)	0.39 ^d^ (±0.02)	Presence of agglomerates
F2	PVA 0.5%	601 ^b^ (±6)	0.33 ^e^ (±0.01)	Presence of agglomerates
F3	Tween^®^ 0.5%	330 (±5)	0.304 (±0.03)	Presence of agglomerates
F4	Pluronic^®^ 1.0%	353.4 (±1.7)	0.198 (±0.01)	Presence of agglomerates
F5	PVA 1.0%	336.1 (±2.3)	0.249 (±0.01)	Homogeneous
F6	Tween^®^ 1.0%	382.1 (±2.9)	0.304 (±0.01)	Homogeneous
F7	Pluronic^®^ 3.0%	322.50 (±0.35)	0.171 (±0.01)	Presence of agglomerates
F8	PVA 3.0%	407.7 (±1.1)	0.204 (±0.02)	Presence of agglomerates
F9	Tween^®^ 3.0%	1283 ^c^ (±85)	0.77 ^c^ (±0.02)	Phase separation

The formulations (F1–F9) were prepared with an aqueous phase containing 10 mL of purified type II water and surfactants (Pluronic^®^ F68, PVA or Tween^®^) at concentrations of 0.5, 1 or 3%, and the lipid phase consisted of stearic acid and oleic acid in a 60:40 ratio followed by sonication for 3 min at 35% amplitude, followed by the addition of 10 mL of purified type II water cooled to 5 °C. F—Formulations. ^a^ F1 vs. F6, F8 (*p* < 0.01). ^b^ F2 vs. all formulations (*p* < 0.0001). ^c^ F9 vs. all formulations (*p* < 0.0001). ^d^ F1 vs. all except F2 (*p* < 0.0001). ^e^ F2 vs. F4, F5, F7 and F8 (*p* < 0.0001). Data were expressed as mean ± standard deviation. Normal distribution was assessed using the Shapiro–Wilk test, adopting a significance level of *p* < 0.05. Statistical analysis was performed using two-way analysis of variance (ANOVA), followed by Tukey’s post hoc test. PVA: poly(vinyl) alcohol.

**Table 2:** Particle diameter and polydispersity index of formulations with different stearic acid:oleic acid proportions.

Formulations	Stearic Acid:Oleic Acid	Particle Diameter	Polydispersity Index
F10	70:30	473.1 (±4.6)	0.35 ^c^ (±0.02)
F11	60:40	336.1 ^a^ (±2.3)	0.25 (±0.01)
F12	50:50	396.8 ^b^ (±3.1)	0.22 (±0.01)

The formulations (F10–F12) were prepared with an aqueous phase containing 10 mL of purified type II water and PVA 1%, the lipid phase consisted of stearic acid and oleic acid in the proportions of 70:30, 60:40 and 50:50, followed by sonication for 3 min at 35% amplitude, followed by the addition of 10 mL of purified type II water cooled to 5 °C. F—Formulations. ^a^ F11 vs. F10, F12 (*p* < 0.0001). ^b^ F12 vs. F10 (*p* < 0.0001). ^c^ F10 vs. F11, F12 (*p* < 0.01). Data were expressed as mean ± standard deviation. Normal distribution was assessed using the Shapiro–Wilk test, adopting a significance level of *p* < 0.05. Statistical analysis was performed using two-way analysis of variance (ANOVA), followed by Tukey’s post hoc test.

**Table 3:** Particle diameter and polydispersity index of formulations varying the sonication time and amplitude.

Formulations	Sonication Amplitude/Time	Particle Diameter	Polydispersity Index
F13	30%/1 min	614.0 ^a^ (±332)	0.66 ^b^ (±0.18)
F14	30%/2 min	404.0 (±209)	0.42 (±0.11)
F15	30%/3 min	238.9 (±9.4)	0.52 (±0.03)
F16	30%/4 min	263.0 (±116)	0.46 (±0.11)
F17	30%/5 min	202.0 (±31)	0.35 (±0.01)
F18	50%/1 min	300.8 (±3.7)	0.39 (±0.03)
F19	50%/2 min	171.9 (±0.9)	0.220 (±0.006)
F20	50%/3 min	170.2 (±1.1)	0.22 (±0.04)
F21	50%/4 min	177.7 (±1.9)	0.27 (±0.03)
F22	50%/5 min	164.9 (±0.32)	0.18 (±0.01)
F23	70%/1 min	299.6 (±2.3)	0.21 (±0.01)
F24	70%/2 min	214.5 (±3.3)	0.222 (±0.007)
F25	70%/3 min	184.5 (±2.7)	0.29 (±0.03)
F26	70%/4 min	261.8 (±3.6)	0.30 (±0.03)
F27	70%/5 min	206.2 (±1.2)	0.34 (±0.02)

The formulations (F13–F27) were prepared with an aqueous phase containing 10 mL of purified type II water and 1% (*w*/*v*) PVA, and the lipid phase consisted of stearic acid and oleic acid in a 60:40 ratio, followed by sonication with times ranging from 1 to 5 min and amplitudes from 30 to 70%, followed by the addition of 10 mL of purified type II water cooled to 5 °C. F—Formulations. Particle diameter—^a^ F13 vs. F17, F19, F20, F21, F22, F24, F25, F27 (*p* < 0.05). Polydispersity Index—^b^ F13 vs. F17, F19, F20, F21, F22, F23, F24, F25, F26 (*p* < 0.0001). Data were expressed as mean ± standard deviation. Normal distribution was assessed using the Shapiro–Wilk test, adopting a significance level of *p* < 0.05. Statistical analysis was performed using two-way analysis of variance (ANOVA), followed by Tukey’s post hoc test.

**Table 4:** Particle diameter and polydispersity index of formulations varying the lecithin concentration.

Formulations	Lectin	Particle Diameter	Polydispersity Index
F28	15 mg	276.0 (±16)	0.341 (±0.008)
F29	25 mg	233.4 (±3.5)	0.25 (±0.01) ^a^
F30	50 mg	269.0 (±15)	0.45 (±0.01)
F31	100 mg	298.0 (±39)	0.50 (±0.14)

The formulations (F28–F31) were prepared with an aqueous phase containing 10 mL of purified type II water and PVA 1%, and the lipid phase consisted of stearic acid and oleic acid in a 60:40 ratio with the addition of lecithin at concentrations of 15, 25, 50 and 100 mg, followed by sonication for 3 min. at an amplitude of 70%, followed by the addition of 10 mL of purified type II water cooled to 5 °C. F—Formulations. ^a^ F29 vs. F31 (*p* < 0.05). Data were expressed as mean ± standard deviation. Normal distribution was assessed using the Shapiro–Wilk test, adopting a significance level of *p* < 0.05. Statistical analysis was performed using two-way analysis of variance (ANOVA), followed by Tukey’s post hoc test.

**Table 5:** The parameters of NLC upon storage up to 90 days.

**NLC-Blank**	**Day 1**	**Day 7**	**Day 14**	**Day 30**	**Day 60**	**Day 90**
Particle diameter	196 ^a^	181 ^a^	180 ^a^	201 ^b^	235	232
	(±1.9)	(±5.2)	(±10)	(±9.2)	(±8.1)	(±3.2)
Polydispersity Index	0.08	0.11	0.09	0.14	0.18	0.27
	(±0.06)	(±0.08)	(±0.10)	(±0.09)	(±0.08)	(±0.09)
Zeta Potential	−17.5	−23.2 ^cf^	−25.0 ^ef^	−24.9 ^e^	−28.4 ^d^	−25.1 ^e^
	(±1.7)	(±0.57)	(±0.97)	(±1.8)	(±1.6)	(±0.47)
**NLC10NOR + 106CN**	**Day 1**	**Day 7**	**Day 14**	**Day 30**	**Day 60**	**Day 90**
Particle diameter	244	250	221 ^b^	216 ^b^	241	349
	(±7.4)	(±7.6)	(±1.7)	(±4.6)	(±4.7)	(±125)
Polydispersity Index	0.32	0.31	0.26	0.14	0.28	0.38
	(±0.00)	(±0.27)	(±0.04)	(±0.11)	(±0.05)	(±0.11)
Zeta Potential	−34.09 ^dgk^	−32.96 ^dhk^	−33.26 ^dghk^	−31.8 ^dgh^	−23.9 ^cij^	−23.9 ^cij^
	(±0.84)	(±0.91)	(±0.85)	(±2.1)	(±2.6)	(±1.5)

Particle diameter—^a^ Day 90 NLC10NOR + 106CN vs. Day 1, 7, 14 NLC-blank (*p* < 0.01); ^b^ Day 90 NLC10NOR + 106CN vs. Day 30 NLC-blank, Day 14, 30 NLC10NOR + 106CN (*p* < 0.05). Zeta Potential—^c^ Day 1 NLC-blank vs. Day 7 NLC-blank, Day 60, 90 NLC10NOR + 106CN (*p* < 0.05); ^d^ Day 1 NLC-blank vs. Day 60 NLC-blank, Day 1, 7, 14, 30 NLC10NOR + 106CN (*p* < 0.0001); ^e^ Day 1 NLC-blank vs. Day 14, 30, 90 NLC-blank (*p* < 0.01); ^f^ Day 1, 7, 14, 30 NLC10NOR + 106CN vs. Day 7, 14 NLC-blank (*p* < 0.0001); ^g^ Day 30 NLC-blank vs. 1, 14, 30 NLC10NOR + 106CN (*p* < 0.001); ^h^ Day 90 NLC-blank vs. 7, 14, 30 NLC10NOR + 106CN (*p* < 0.001); ^i^ Day 7 NLC10NOR + 106CN vs. day 60, 90 NLC10NOR + 106CN (*p* < 0.001); ^j^ Day 30 NLC10NOR + 106CN vs. 60, 90 NLC10NOR + 106CN (*p* < 0.01); ^k^ Day 60, 90 NLC10NOR + 106CN vs. Day 1, 7, 14 NLC10NOR + 106CN (*p* < 0.0001). Data were expressed as mean ± standard deviation. Normal distribution was assessed using the Shapiro–Wilk test, adopting a significance level of *p* < 0.05. Statistical analysis was performed using two-way analysis of variance (ANOVA), followed by Tukey’s post hoc test. NLC-blank—Drug-free nanostructured lipid carrier. NLC10NOR + 106CN—Nanostructured lipid carrier with 10 mg of norfloxacin and 10 mg of 6CN-Ethyl.

## Data Availability

The original contributions presented in this study are included in the article and Supplementary Materials. Further inquiries can be directed to the corresponding author.

## Supplementary Materials

The following supporting information can be downloaded at: https://www.mdpi.com/article/10.3390/pharmaceutics18020183/s1, Figure S1: Calibration curve of peak area versus concentration for norfloxacin (278 nm) and 6CN-Ethyl (221 nm) obtained by HPLC.

## Abbreviations

The following abbreviations are used in this manuscript:

ATR	Attenuated Total Reflectance
BHI	Brain Heart Infusion
CCCP	Carbonyl Cyanide m-Chlorophenylhydrazone
CFU	Colony Forming Units
DLS	Dynamic Light Scattering
DSC	Differential Scanning Calorimetry
DMSO	Dimethyl Sulfoxide
EE%	Encapsulation Efficiency
ESKAPE	*Enterococcus faecium*, *Staphylococcus aureus*, *Klebsiella pneumoniae*, *Acinetobacter baumannii*, *Pseudomonas aeruginosa*, *Enterobacter* spp.
FTIR	Fourier Transform Infrared Spectroscopy
MATE	Multidrug and Toxic Compound Extrusion
MDR	Multidrug Resistance
MepA	Multidrug Efflux Protein A
MRSA	Methicillin-Resistant *Staphylococcus aureus*
MIC	Minimum Inhibitory Concentration
MFS	Major Facilitator Superfamily
M.p.	Melting point
NLCs	Nanostructured Lipid Carriers
NOR	Norfloxacin
NorA	NorA efflux pump
PDI	Polydispersity Index
PVA	Polyvinyl Alcohol
*S. aureus*	*Staphylococcus aureus*
SEM	Standard Error of the Mean
XRD	X-ray Diffraction
ZP	Zeta Potential
