# Supplementary material for: Nanostructured Lipid Carriers Containing Norfloxacin and 2-Aminothiophene Derivative Reduces Fluoroquinolone Resistance in Multidrug-Resistant Staphylococcus aureus Strains by Efflux Pump Inhibition
Source: Pharmaceutics. 2026 Jan 30;18(2):183. doi: 10.3390/pharmaceutics18020183 (PMC12944099; doi:10.3390/pharmaceutics18020183)
Supplement: Supplementary file 1 [file pharmaceutics-18-00183-s001.zip › pharmaceutics-4059539-supplementary.pdf]

pharmaceutics

MDPI

# SUPPLEMENTARY MATERIAL

## Nanostructured Lipid Carriers Containing Norfloxacin and 2-Aminothiophene Derivative Reduces Fluoroquinolone Resistance in Multidrug-Resistant Staphylococcus aureus Strains by Efflux Pump Inhibition

**Aléxia Gonçalves Dias [1], Izabele de Souza Araújo [1], Rodrigo Santos Aquino de Araújo [2], Malu Maria Lucas dos Reis [2], Cícera Datiane de Morais Oliveira Tintino [3], Saulo Relison Tintino [3], Gildênia Alves de Araújo [3], Priscilla Augusta de Sousa Fernandes [1], Henrique Douglas Melo Coutinho [3], Elquio Eleamen Oliveira [2,*] and Francisco Jaime Bezerra Mendonça-Junior [2]**

[1] *Postgraduate Program in Natural and Synthetic Bioactive Products, Federal University of Paraíba, João Pessoa 58051-900, Brazil; alexiajgdias@gmail.com (A.G.D.); izabele.araaujo@gmail.com (I.d.S.A.); prisciasf@gmail.com (P.A.d.S.F.)*

[2] *Laboratory of Synthesis and Drug Delivery, Department of Biological Sciences, State University of Paraíba, João Pessoa 58071-160, Brazil; rodrigobiologojp@gmail.com (R.S.A.d.A.); malureisduarte@gmail.com (M.M.L.d.R.); franciscojaime@servidor.uepb.edu.br (F.J.B.M.-J.)*

[3] *Laboratory of Microbiology and Molecular Biology, Universidade Regional do Cariri, Crato 63105-000, Brazil; datianemorais@gmail.com (C.D.d.M.O.T.); saulo.tintino@urca.br (S.R.T.); gildenia.araujo@urca.br (G.A.d.A.); hdmcoutinho@gmail.com (H.D.M.C.)*

* Correspondence: *elquioeleamen@servidor.uepb.edu.br; Tel.: +55-84-9987-7523*

## SUPPLEMENTARY MATERIAL

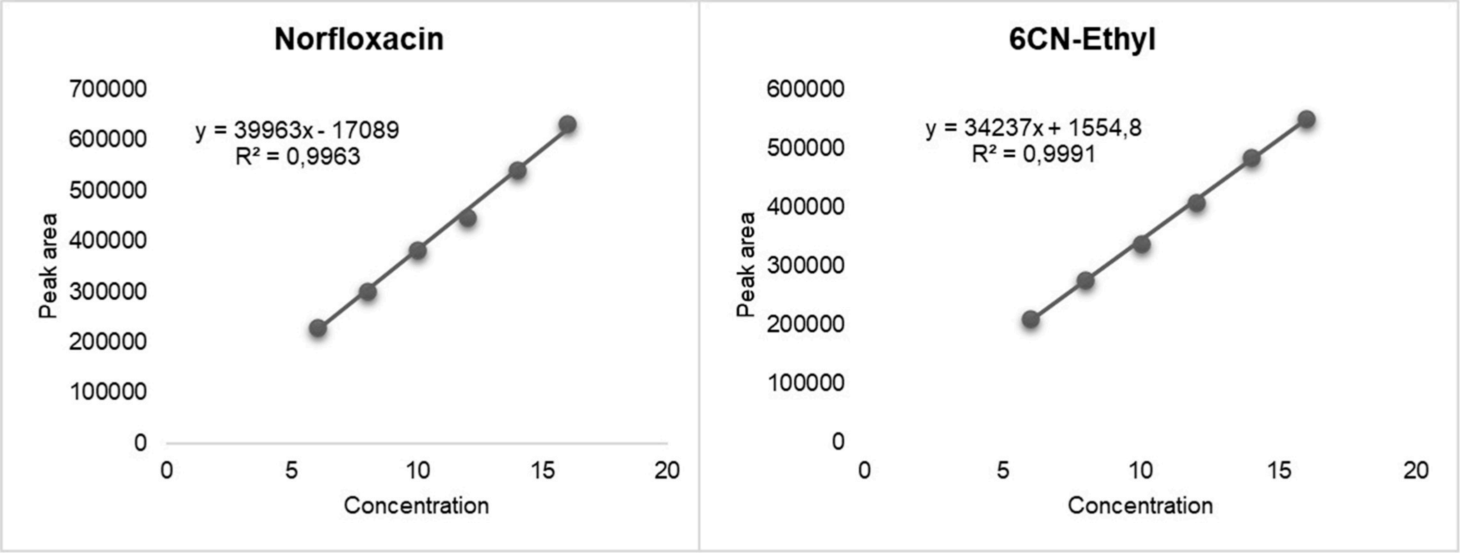


**Figure S1:** Calibration curves of peak area versus concentration for norfloxacin (278 nm) and 6CN-Ethyl (221 nm) obtained by HPLC.
